# Evaluating the resistance pattern of gram-negative bacteria during three years at the nephrology ward of a referral hospital in southwest of Iran

**DOI:** 10.15171/jnp.2017.35

**Published:** 2017-04-02

**Authors:** Iman Karimzadeh, Niloofar Sadeghimanesh, Mona Mirzaee, Mohammad Mahdi Sagheb

**Affiliations:** ^1^Department of Clinical Pharmacy, Faculty of Pharmacy, Shiraz University of Medical Sciences, Shiraz, Iran; ^2^Nephrology-Urology Research Center and Department of Internal Medicine, Shiraz University of Medical Sciences, Shiraz, Iran

**Keywords:** Antibacterials, Drug resistance, Gram-negative bacteria, Nephrology

## Abstract

**Background:**

Gram-negative bacteria are associated with an increase in rates of antibacterial resistance. In most low- and middle-income countries such as Iran, there is no continuous surveillance system for antibiotic resistance.

**Objective:**

The purpose of this survey was to determine the pattern of antimicrobial sensitivity of gram-negative bacteria within 3 consecutive years at a nephrology ward of Nemazee hospital in Shiraz.

**Materials and Methods:**

During a 3-year period from 2013 to 2015 at the adult nephrology ward, bacteriological data of all biological samples of hospitalized patients in favor of gram-negative microorganisms were analyzed retrospectively. Antimicrobial susceptibility was performed by the Kirby-Bauer disc diffusion method.

**Results:**

The most common gram negative bacterium isolated from biological samples was Escherichia coli (43.9%). The highest (86.3%-94.1%) antibacterial resistance rate was associated with Acinetobacter spp. The most frequent resistance was seen with cephalosporins. In contrast to ceftriaxone, ciprofloxacin, and trimethoprim/sulfamethoxazole, nitrofurantoin and aminoglycosides remained their acceptable activity against E. coli. At least three-fourths (75%) of Acinetobacter spp. isolates was resistant to either aminoglycosides or imipenem. All (100%) isolated Acinetobacter spp. and Pseudomonas aeruginosa species were susceptible to colistin. The rate of Acinetobacter spp. and P. aeruginosa resistant to three or more drugs was 81.7% and 74.6%, respectively.

**Conclusions:**

The resistant rate of gram negative pathogens to different tested antibacterial agents was considerably high and has increased during the recent three years in our center.

**Implication for health policy/practice/research/medical education:**

Antibiotic resistance is responsible for considerable morbidity and mortality worldwide. Most low- and middle-income countries such as Iran lack national continuous surveillance systems for antibiotic resistance. Resistant rate of gram-negative pathogens to different antibacterial agents especially cephalosporins are considerably high in the nephrology ward. Nitrofurantoin and aminoglycosides remained their acceptable activity against Escherichia coli during the recent 3 years in the nephrology ward. Resistant rate of gram-negative pathogens especially Acinetobacter spp. have increased for most tested antibacterial agents during the recent 3 years in the nephrology ward.

## 1. Background

Infections caused by antibiotic resistant bacteria are a significant cause of morbidity and mortality worldwide (1). According to the Centers for Disease Control and Prevention (CDC) estimation, antibiotic resistance is responsible for more than 2 million infections and 23 000 deaths each year in the United States (2). It has been also estimated that more than 70% of the bacteria that causes hospital acquired infections are resistant to at least one antibiotic (3).

Infectious diseases are common cause of morbidity and considered as the second cause of mortality in chronic kidney disease patients (4). Sepsis-related death among hemodialysis patients is 100 times more than the general population (5). On the other hand, urinary, respiratory, and hemodialysis catheter-related bloodstream infections are common among patients with renal diseases (4).

In line with gram-positive pathogens (mainly *Staphylococcus aureus* and *Staphylococcus epidermidis*), gram-negative bacteria are also associated with an increase in rates of antibacterial resistance in catheter-related infections. Among gram negatives, extended-spectrum β-lactamase (ESBL), carbapenem-resistant, and multi-drug resistant Enterobacteriaceae specially pose a serious threat to patients in healthcare settings (6). Most low- and middle-income countries such as Iran lack national continuous surveillance systems for antibiotic resistance. Having awareness of antimicrobial resistance pattern can lead to selecting an optimized antimicrobial agent or regimen and consequently, minimizing duration of hospitalization, morbidity, mortality, and direct as well as indirect health care costs (7).

## 2. Objectives

The purpose of the current study was to determine the plausible changing pattern of antimicrobial sensitivity of gram negative bacteria within 3 consecutive years at a nephrology ward of a referral teaching hospital in Shiraz, Southwest of Iran.

## 3. Materials and Methods

A retrospective study was conducted on microbiological data of hospitalized patients between 2013 and 2015 at a 20-bed adult nephrology ward of Nemazee hospital affiliated to Shiraz University of Medical Sciences.

Bacteriological data of all biological isolates including blood, urine, sputum, wound drainage, abscess, synovial, pleural, ascitic, tip of catheter, and cerebrospinal fluid taken either before as empirical therapy or within antibacterial treatment as definite therapy of both community and hospital-acquired infections sent to the laboratory of the hospital were collected. Except for hospitalization at the nephrology ward during the study period, no specific inclusion–exclusion criteria were implemented for patient recruitment.

Identification of bacteria was done by the initial gram staining and additional biochemical tests such as nitrate, indole, oxidase, urease, and DNase. Mueller-Hinton agar culture medium (Merck, Germany) was inoculated with a saline suspension of isolated aerobic gram negative bacteria equivalent to McFarland 0.5 turbidity standards. Antibiotic discs (Padtan Teb Co, Iran) were applied on the surface of agar. After 16-18 hours incubation at 37°C, the antimicrobial susceptibility of gram-negative bacteria was determined by the Kirby-Bauer disc diffusion method based on the Clinical and Laboratory Standard Institute (CLSI) guideline (8). The isolated aerobic gram-negative bacteria were categorized to be resistant, intermediately resistant, or sensitive regarding the size of inhibition zone. The following antibiotic discs (per unit disc) were used for antimicrobial susceptibility of isolated gram-negative bacteria: cephalexin (30 µg), ceftriaxone (30 µg), cefotaxime (30 µg), ceftizoxime (30 µg), ceftazidime (30 µg), cefixime (5 µg), trimethoprim/sulfamethoxazole [25 (1.25/23.75) µg], gentamicin (10 µg), amikacin (30 µg), ciprofloxacin (5 µg), nitrofurantoin (300 µg), chloramphenicol (30 µg), imipenem (10 µg), and colistin (10 µg). Isolates simultaneously resistant to 2 or more drugs were considered as multidrug resistant (MDR).

### 3.1. Ethical issues

1) The research followed the tenets of the Declaration of Helsinki; and 2). The study was approved by the Institutional Review Board (IRB) and the Medical Ethics Committee of the Nemazee hospital related to Shiraz University of Medical Sciences.

### 3.2. Statistical analysis

Continuous and categorical variables were expressed as mean ± standard deviation (SD) and percentage, respectively. Descriptive analyses were carried out by SPSS (Statistical Package for the Social Sciences) version 20 software.

## 4. Results

A total number of 305 gram-negative bacteria (109, 100, and 96 isolates in 2013, 2014, and 2015, respectively) were isolated from 252 patients (89, 83, and 80 subjects in 2013, 2014, and 2015, respectively). The mean ± SD of the study population was 53.1 ± 19.3 years. One hundred three and 149 patients were male and female, respectively. The 3 most common isolated gram-negative bacteria from all biological samples of patients were *Escherichia coli* (43.9%), *Klebsiella* spp*.* (19.3%), and *Acinetobacter* spp. (15.4%). Most gram-negative bacteria were isolated from urine (57.1%) followed by blood (19.7%) (Table 1).

**Table 1:** Frequency of gram-negative bacteria isolated from different biological specimens of patients hospitalized at the nephrology ward (n = 305)

** Microorganism/Sample**	** Blood (n)**	** Urine (n)**	** Sputum (n)**	Other* (n)
*Klebsiella* spp.	16	28	7	8
*Escherichia coli*	18	104	4	8
*Acinetobacter* spp.	8	14	16	9
*Enterobacter* spp.	11	13	5	5
*Citrobacter* spp.	-	4	-	1
*Pseudomonas aeruginosa*	5	10	3	4
*Stenotrophomonas maltophilia *	2	1	1	-
Total	60	174	36	35
Percent	19.7	57.1	11.8	11.5

*Including abscess, synovial, pleural, ascitic, tip of catheter, and cerebrospinal fluids.

Tables 2 and 3 summarize the antimicrobial resistance pattern of isolated gram-negative bacteria to different antibiotics during 3 consecutive years. Among identified gram-negatives, *Acinetobacter* spp. and *Enterobacter* spp. were associated with the highest (86.3%-94.1%) and lowest (9.5%-44.7%) antibacterial resistance rates, respectively. Regarding antibacterial agents studied, the most frequent resistance was seen with cephalosporins including cephalexin (80-95.7%), cefixime (70.6%-89.2%), ceftriaxone (67.6%-94.9%), cefotaxime (70.4%-100%), and ceftazidime (75%-100%). The 3 antibacterial agents with the lowest resistant rates were colistin (4.76%), amikacin (27.3%-35 %), and chloramphenicol (41.2%-52.3%).

**Table 2:** Sensitivity, intermediate resistance, and resistance frequency of isolated gram negative bacteria to different tested antimicrobials during 2013, 2014, and 2015 at the nephrology ward

** Antimicrobial agent/Microorganism**	** Sensitive (n) **			**Intermediate (n)**			** Resistant (n)**		
	**2013 **	**2014 **	**2015 **	**2013**	**2014 **	**2015 **	**2013 **	**2014**	** 2015**
Trimethoprim/Sulfamethoxazole									
Number	22	35	10	-	2	1	45	74	31
Percent	32.8	31.5	23.8	-	1.8	2.4	67.2	66.7	73.8
Amikacin									
Number	51	52	19	25	25	13	41	35	12
Percent	43.6	46.4	43.2	21.4	22.3	29.6	35	31.3	27.3
Gentamicin									
Number	20	62	17	1	3	2	28	49	23
Percent	40.8	54.4	40.5	2	2.6	4.8	57.1	42.9	54.8
Ciprofloxacin									
Number	46	51	15	1	1	4	66	67	28
Percent	40.7	42.9	31.9	0.9	0.8	8.5	58.4	56.3	59.6
Nitrofurantoin									
Number	4	39	15	1	13	3	33	26	5
Percent	10.5	50	65.2	2.6	16.7	13	86.8	33.3	21.7
Chloramphenicol									
Number	18	24	9	3	-	1	23	17	7
Percent	40.9	58.5	52.9	6.8	-	5.9	52.3	41.5	41.2
Cephalexin									
Number	2	20	6	-	2	2	44	82	32
Percent	4.4	19.2	15	-	1.9	5	95.7	78.9	80
Cefixime									
Number	12	31	4	3	1	-	-	77	33
Percent	80	28.4	10.8	20	0.9	-	-	70.6	89.2
Ceftriaxone									
Number	-	23	6	2	1	-	37	50	20
Percent	-	31.1	23.1	5.1	1.4	-	94.9	67.6	76.9
Cefotaxime									
Number	-	8	8	-	-	-	1	32	19
Percent	-	20	29.6	-	-	-	100	80	70.4
Ceftazidime									
Number	-	-	-	-	1	-	-	3	4
Percent	-	-	-	-	25	-	-	75	100
Ceftizoxime									
Number	6	29	3	-	6	2	37	37	12
Percent	13.9	40.3	17.7	-	8.3	11.8	86.1	51.4	70.6
Imipenem									
Number	5	4	11	1	-	1	6	6	13
Percent	41.7	40	44	8.3	-	4	50	60	52
Colistin									
Number	-	3	19	-	-	1	-	-	1
Percent	-	100	90.5	-	-	4.8	-	-	4.8
Total number	186	381	142	37	55	30	361	555	240
Total percent	31.9	38.5	34.5	6.3	5.6	7.3	61.8	56	58.3

**Table 3:** Sensitivity, intermediate resistance, and resistance frequency of some isolated gram negative bacteria to different tested antimicrobials during 2013, 2014, and 2015 at the nephrology ward

** Antimicrobial agent/Microorganism**	** Sensitive (n) **			** Intermediate (n)**			** Resistant (n)**		
	** 2013 **	** 2014 **	**2015 **	**2013 **	**2014 **	**2015**	** 2013**	** 2014 **	**2015**
* Escherichia coli *									
Trimethoprim/Sulfamethoxazole	8	14	6	-	-	-	5	42	17
Ciprofloxacin	17	18	7	1	1	1	41	38	16
Chloramphenicol	8	10	5	3	-	-	1	1	1
Nitrofurantoin	-	35	15	-	10	2	12	6	-
Amikacin	- 27	12	24	18	7	11	13	4
Gentamicin	-	39	12	-	3	-	-	20	10
Imipenem	-	1	3	1	-	-	2	2	3
Colistin	-	-	6	-	-	-	-	-	-
Cefixime	12	15	5	1	1 -	-	39	18
Ceftriaxone	-	13	5	1	-	-	36	34	14
Ceftizoxime	-	13	-	-	4	-	37	24	4
Ceftazidime	-	-	-	-	-	-	-	1	-
Cefotaxime	-	5	5	-	-	-	1	13	12
Cephalexin	-	10	4	-	1	2	43	40	17
Total	45	200	101	31	38	12	189	273	116
Percent	16.9	39.1	44.1	11.7	7.4	5.2	71.3	53.4	50.7
* Klebsiella * spp.									
Trimethoprim/Sulfamethoxazole	7	12	2	-	-	-	12	11 5
Ciprofloxacin	11	15	4	-	-	2	8	8	3
Chloramphenicol	9	6	3	-	-	-	2	3	-
Nitrofurantoin	2	1	-	2	2	1	7	11	3
Amikacin	8	12	3	-	4	5	8	6	1
Gentamicin	15	16	2	-	- 1	5	8	4
Imipenem	4	-	2	-	-	1	-	1	2
Colistin	-	-	3	-	-	-	-	-	-
Cephalexin	-	8	1	-	-	-	-	15	6
Cefixime	-	9	2	-	-	-	-	12	5
Ceftizoxime	-	9	1	-	1	2	-	-	3
Ceftriaxone	-	5	1	-	1	-	-	7	3
Cefotaxime	-	2	1	-	-	-	-	10	2
Ceftazidime	-	-	-	-	-	-	-	1	-
Total	56	96	25	2	8	12	42	93	37
Percent	56	48.7	33.8	2	4.1	16.2	42	47.2	50
* Acinetobacter * spp.									
Trimethoprim/Sulfamethoxazole	-	-	-	-	-	1	19	10	6
Ciprofloxacin	4	-	-	-	-	1	15	14	7
Nitrofurantoin	-	-	-	-	-	-	7	5	-
Chloramphenicol	-	-	-	-	-	-	13	8	5
Amikacin	-	1	-	-	-	-	13	11	7
Gentamicin	1	1	-	1	-	-	16	12	7
Imipenem	1	-	-	-	-	-	3	3	7
Colistin	-	2	7	-	-	-	-	-	-
Ceftazidime	-	-	-	-	-	-	-	2	3
Cefixime	-	-	-	-	-	-	-	14	7
Ceftizoxime	-	1	-	-	-	-	-	6	4
Cefotaxime	-	-	-	-	-	-	-	6	1
Ceftriaxone	-	1	-	-	-	-	-	5	2
Total	6	6	7	1	-	2	86	96	56
Percent	6.5	5.9	10.8	1.1	-	3.1	92.5	94.1	86.2
* Pseudomonas aeruginosa *									
Trimethoprim/Sulfamethoxazole	-	-	-	-	-	-	6	4	3
Ciprofloxacin	6	5	2	-	-	-	2	1	1
Nitrofurantoin	-	-	-	-	-	-	4	2	1
Chloramphenicol	-	-	-	-	-	-	4	2	1
Amikacin	-	5	1	-	-	1	5	1	-
Gentamicin	-	4 1	-	-	-	4	1	2
Imipenem	-	-	2	-	-	-	1	-	-
Colistin	-	-	2	-	-	1	-	-	-
Ceftriaxone	-	1	-	1	-	-	-	1	1
Cefixime	-	-	-	-	-	-	-	4	3
Cefotaxime	-	-	-	-	-	-	-	1	1
Ceftizoxime	-	1	-	-	1	-	-	2	1
Total	6	16	8	1	1	2	26	19	14
Percent	18.2	44.4	33.3	3	2.8	8.3	78.8	52.8	58.3
* Enterobacter * spp.									
Trimethoprim/Sulfamethoxazole	5	4	2	-	2	-	5	7	-
Ciprofloxacin	7	8	2	2	-	-	4	4	-
Nitrofurantoin	-	1	-	-	1	-	3	-	1
Chloramphenicol	2	6	1	2	1	-	6	-	-
Amikacin	6	4	2	1	2	-	4	2	-
Gentamicin	7	6	2	-	-	-	3	6	-
Imipenem	-	2	1	-	-	-	-	-	-
Colistin	-	1	1	-	-	-	-	-	-
Cefixime	3	6	2	2	-	-	5	5	-
Ceftizoxime	6	4	1	-	-	-	3	-	-
Ceftazidime	-	-	-	-	1	-	1	-	-
Cefotaxime	1	-	1	-	-	-	-	1	-
Ceftriaxone	3	2	1	-	-	-	1	1	-
Total	40	44	16	7	8	-	35	26	1
Percent	48.8	56.4	94.1	8.5	10.3	-	42.7	33.3	5.9

Regarding *E. coli*, the rate of ceftriaxone resistance in 2013, 2014, and 2015 were 97.2%, 72.3%, and 73.7%, respectively. Ciprofloxacin-resistant *E. coli* was 69.5% in 2013. Interestingly, its rates were 66.7% in both 2014 and 2015. The rate of *E. coli* resistant to trimethoprim/sulfamethoxazole increased from 62.5% in 2013 to 73.9% in 2015. All (100%) *E. coli* isolates were resistant to nitrofurantoin in 2013. However, the rate of nitrofurantoin-resistant *E. coli* decreased to 11.8%, and 0% in 2014 and 2015, respectively. Frequency of *E. coli* resistant to aminoglycosides including amikacin and gentamicin ranged between 27.5% and 31.4% during the study period.

The rate of imipenem-resistant *Klebsiella* spp*.* in 2013, 2014, and 2015 was 0%, 100%, and 40%, respectively. All isolates of this bacteria was sensitive to colistin in 2015. Aminoglycoside-resistant *Klebsiella* spp*.* decreased from 36.1% in 2013 to 31.3% in 2015. Similarly, the frequency of *Klebsiella* spp*.* resistant to ciprofloxacin decreased from 42.1% in 2013 to 33.3% in 2015. Between 52.6% and 65% of *Klebsiella* spp*.* isolates were resistant to all tested third-generation cephalosporins within the study period.

All (100%) isolates of *Acinetobacter* spp. were susceptible to colistin within the study. In contrast, all *Acinetobacter* spp. were resistant to chloramphenicol. The rates of imipenem-resistant *Acinetobacter* spp. in 2013, 2014, and 2015 were 75%, 100%, and 100%, respectively. Frequency of aminoglycosides (including amikacin and gentamicin) resistance *Acinetobacter* spp. increased from 93.6% in 2013 to 100% in 2015. More than one-fifth (21.1%) of *Acinetobacter* spp. isolates were susceptible to ciprofloxacin in 2013. However, all *Acinetobacter* spp. isolates became resistant to this agent in 2014 and 2015. Resistance to three or more drugs was observed in 81.7% of isolated *Acinetobacter* spp. during the study.

In 2015, all (100%) isolates of *Pseudomonas aeruginosa* were sensitive to both colistin and imipenem. Frequency of *P. aeruginosa* resistant to aminoglycosides including amikacin and gentamicin decreased from 100% in 2013 to 40% in 2015. In contrast, the rate of ciprofloxacin-resistant *P. aeruginosa* increased from 25% in 2013 to 50% in 2015. The resistance of this pathogen to both trimethoprim/sulfamethoxazole and chloramphenicol was 100% within 3 years. During the study period, 74.6% of isolated *P. aeruginosa* were considered as MDR.

## 5. Discussion

The most frequent gram-negative pathogen in our survey was* E. coli*. This is in accordance with a European and North American surveillance study on more than 220 000 samples collected from intensive care units during 2000-2002 (9). A cross-sectional study on 86 patients with infected diabetic foot admitted to Nemazee hospital from July 2011 to June 2012 demonstrated *E. coli* as the most common gram-negative bacteria (10). Similar findings were also reported by Khalili et al (11) and Moradi et al (12) from 2 teaching hospitals at Tehran and Bandar Abbas in Iran, respectively.

Among studied antibacterial agents, the most frequent resistance in our population was seen with cephalosporins. Regarding ceftazidime as the only current third generation cephalosporin active against Pseudomonas for example, between 75% and 100% of all isolated gram-negative pathogens were demonstrated to be resistant to this agent in the present study. In contrast, antimicrobial resistance surveillance study at 100 medical centers in Japan in 2004 demonstrated that only 6% of *Acinetobacter* spp. and 9.9% of *P. aeruginosa* isolates were resistant to ceftazidime (13). The rate of ceftazidime-resistant *Acinetobacter baumannii* at Saudi Arabia in 2011 was reported to be 89% (14). At least one study from a hospital in Tehran also reported that cefixime, cefotaxime, ceftriaxone, and ceftazidime were associated with the highest rate of resistance of isolated gram-negative bacteria (11). Hadadi et al demonstrated that more than two-thirds (76.5%) of these pathogens from 2 university hospitals in Iran were resistant to ceftazidime (15). The resistant rate of gram-negative bacteria to ceftazidime isolated from patients with ICU-acquired infections in 2 studies at Shiraz ranged between 57.9% (16) and 61.5% (17). These findings may be due to antimicrobial selection pressure caused by overuse of this class of antibiotic. In this regards, according to the National Rational Drug Use Committee official report, cefixime has been ranked as the eighth, sixth, and fifth commonly prescribed medications by physicians in Iran at 2008, 2009, and 2010, respectively (18). Availability, low rate of side effects, and relatively low cost partially can be taken into account this issue (15).

Although variable and even decreased in the case of ceftriaxone during 3 years, but the rate of *E. coli* resistant to commonly used antibiotics for this pathogen in the setting of urinary tract infection including ceftriaxone (72.3%-97.2%), ciprofloxacin (66.7%-69.5%), and trimethoprim/sulfamethoxazole (62.5%-73.9%) was relatively high in our study. Similarly, Hadadi et al reported that 68% and 72% of isolated *E. coli* were resistant to ceftriaxone and ciprofloxacin, respectively (15). In contrast to these data, the frequency of ciprofloxacin-resistant* E. coli* in at least 2 Karaj (19) and Tehran (Imam Khomeini) (11) hospitals was relatively low (from 26.3% to 54.5%). These variations can be partially explained by the fact that fluoroquinolones may have not been routinely used as an empirical antibiotic for infectious diseases in hospitals with favorable susceptibility rates to these agents. Although not tested in our survey, the resistant pattern of *E. coli* to ciprofloxacin can be extrapolated to levofloxacin, as a new commonly prescribed agent by most physicians for the treatment of urinary tract infections in Iran, due to possible cross-resistance within fluoroquinolones (20).

Nitrofurantoin is highly effective against *E. coli* with relatively low rates of resistance (<10%) in most geographic areas of the world (21). Two studies from Karaj and Ahvaz reported that the rates of *E. coli* susceptibility to nitrofurantoin were 90.8% (19) and 71.2% (22), respectively. Japoni et al survey on 200 *E. coli* isolates from 3 hospitals and 2 health centers affiliated to Shiraz University of Medical Sciences demonstrated the resistant rate of 25% to nitrofurantoin (23). *E. coli* strains recovered from urine samples of children with community-acquired urinary tract infections in Jahrom were susceptible to nitrofurantoin in 96.8% of cases (24). The rate of nitrofurantoin-resistant *E. coli* decreased considerably within 3 years in the current study. Although the rate of *E. coli* susceptibility to nitrofurantoin was relatively acceptable in our clinical setting, this agent seems not to be a favorable antibacterial in nephrology wards. This may be due to fact that most of admitted patients have impaired renal function and administering nitrofurantoin is not generally recommended in individuals with creatinine clearance less than 60 or 40 mL/min in spite of controversies and lack of enough clinical data in this regard (25).

The similar story appears to exist for aminoglycosides. The resistance rates of *E. coli* to amikacin and gentamicin in the present study were only 4%-11% and 10%-20%, respectively. In addition, the resistant rate of all our isolated gram-negative bacteria to amikacin was 27.3%-35% and to gentamicin was 42.9%-57.1%. In agreement with our data, Khalili et al implicated that only 21.3%-33.3% of gram-negative pathogens during a four-year period were resistant to amikacin at a referral infectious disease ward in Tehran (11). This ranged from 0% to 21.4% at a teaching hospital in Urmia (26). Poorabbas et al reported that with the exception of *Acinetobacter* spp., other gram-negative pathogens isolated from seven teaching hospitals in Iran exhibited susceptibility rate of at least 60% to amikacin (27). Investigations over a long time period in other countries such as the United States and Taiwan have also revealed that an increase in the rate of resistance to aminoglycosides has been somewhat milder compared to other antibacterial agents (28,29). Despite its relative favorable susceptibility pattern in our ward, nephrologists generally do not prefer aminoglycosides as drugs of choice for treatment of gram-negative infections (e.g., *E. coli*) due to the presence of an underlying kidney disease in admitted patients and also the nephrotoxic as well as ototoxic properties of these agents. Lack of routine therapeutic drug monitoring for aminoglycosides in our center can partially justify clinician concerns on this issue.

Except for colistin, the resistant rate of *Acinetobacter* spp. to other studied antibacterials during the study period was considerably high. This is especially concerning for imipenem because carbapenems were previously considered as the most effective antibacterial agent for the treatment of *Acinetobacter* spp. infections. The frequency of resistance to imipenem was reported to be 13%, 8%, and 33.4% in Belgium (30), Polish (31), and Turkey (32), respectively. During the recent decade in Iran, the frequency of carbapenem-resistant *Acinetobacter* spp. has been increased. It has been ranged from only 14.6% in 2004-2005 to 50.9%, 52.5%, 62%, 67.5%, 91.5%, and 99% in years between 2008 and 2013 at Tehran hospitals (33). This rate reported from other regions of Iran has been 60.8% in Bandar Abbas at 2006 (12), 96.1% in Ahvaz at 2013 (34), and 75%-79.8% in Kermanshah at 2013 (35). According to a multicentre collaborative study within 2008-2009 on seven major teaching hospitals in Shiraz, Tabriz, Sari, Mashhad, Sanandaj, Ahvaz, and Isfahan, 36% of *Acinetobacter* strains were resistant or intermediate- resistant to imipenem (27). In Shiraz, a survey on 836 adult patients admitted to ICUs at the Nemazee hospital during 2008 and 2009 demonstrated that only 20% of *Acinetobacter* spp. isolates were resistant to either imipenem or meropenem (16). A newer study on four Shiraz teaching hospitals at 2013 reported 98.5% and 99.5% as the rate of imipenem- and meropenem-resistant *Acinetobacter* spp*.* (33).

Apart from carbapenem resistance, 75.6% of *Acinetobacter* spp. isolates in our survey were considered to be MDR. The rate of MDR among *A. baumannii* strains was 27.6% to 32.5% in non-ICU inpatients isolates and 11.6% to 24.2% from ICU inpatients from a study in the United States (36). In a survey from 2000 to 2002 at a university hospital in the neighbor country, Turkey, MDR was reported in 45.4% and 37.7% of *Acinetobacter* spp. and *P. aeruginosa* isolates, respectively (37). This rate was reported to be 81.7% at 2 hospitals in Tehran (15) and 95% in Isfahan (38) within the ICU setting. These data highlight the concern over a real threat of untreatable *Acinetobacter* spp. infections. Inappropriate utilization pattern along with overuse of agents with activity against gram-negative bacteria especially carbapenems for the treatment of both community and hospital acquired infections, as shown in at least 2 studies from Sari (39) and Tehran (40), appear to be the main cause of high burden of *Acinetobacter* spp. resistant to carbapenems in our country. Interestingly, Sistanizad et al demonstrated that implementing a restriction policy through prescribing carbapenems only for infections caused by culture proven multi-drug-resistant gram-negative bacteria only sensitive to these agents resulted in a significant increase in the sensitivity rate of *P*. *aeruginosa* to imipenem at ICU after 6 months (41).

All isolated *Acinetobacter* spp. and *P. aeruginosa* species in our ward were susceptible to colistin. Low rates of colistin-resistant *Acinetobacter* spp. have been reported in our country. For example, 1% of *Acinetobacter* spp. isolates at Hamedan in 2011-2012 (42), 11.6% at Isfahan in 2011-2012, (38) and 12% at Tehran in 2009-2010 (43) were resistant to colistin. However, most recent studies from different parts of Iran including Tehran (44), Ahvaz (34), and Kermanshah (35) revealed that the susceptible rate of *Acinetobacter* spp. to colistin has been 100%. Similar findings have been reported from at least 2 surveys in Shiraz including Nemazee hospital in 2008-2009 (16) and 2013 (33). Therefore, this agent in combination with other appropriate antibiotics can be considered as the last antibacterial resort for treatment of infections caused by MDR gram-negative pathogens in our center. However, noting that routine use of colistin antibiogram for Enterobacteriaceae isolates has been initiated only in limited wards of Nemazee hospital from 2014. Thus, our data in this regard need validation with new, long enough surveys.

## 6. Conclusions

Our findings implicated that resistant rate of gram-negative pathogens to different antibacterial agents especially cephalosporins are considerably high and increased during the recent years. At least 50% of isolated gram-negative bacteria were resistant to imipenem. Among gram-negative isolates, *Acinetobacter* spp. is associated with the highest rate of resistance. Except for *Acinetobacter* spp., the resistant rate of all isolated gram-negative isolates to tested aminoglycosides appears to be relatively favorable. Performing regular and periodic surveillance of antimicrobial resistance pattern by the comprehensive, multi-specialty infection control committee appear to have a key role in optimizing both the empirical and definite antibacterial treatment regimens and may also slow down the runaway train of antibacterial resistance at our clinical settings in Iran.

## Limitations of the study

The present study suffers from 3 major limitations. First, the possible association of patients’ antibiogram with their response to antimicrobial treatment in real clinical conditions could not be assessed due to the retrospective pattern of the study. Second, antimicrobial susceptibility test was performed by the classic disc diffusion method rather than more accurate methods such as microbroth dilution or E-test. Third, some antibacterial agents with defined activity against gram-negative bacteria including piperacillin/tazobactam, cefepime, ofloxacin, levofloxacin, and gemifloxacin currently available in Iran were not tested by the hospital laboratory during the study period and their susceptibility rates remain unknown.

## Acknowledgments

This study was the result of a research project (# 94-01-05-9862) approved and supported by a Vice-Chancellor for Research of Shiraz University of Medical Sciences. Authors would like to acknowledge the Nemazee Hospital Central Laboratory manager and staffs for their kind cooperation and support.

## Authors’ contribution

IK contributed to study design, data analysis, and manuscript review. MM contributed to data collection and manuscript drafting. NS contributed to data collection and manuscript drafting. MMS contributed to clinical interpretation of data and manuscript review.

## Conflicts of interest

There were no points of conflicts.

## Funding/Support

None.

## References

[1] World Health Organization (WHO). The top 10 causes of death. http://www.who.int/mediacentre/factsheets/fs310/en/. Accessed on March 13, 2016.

[2] Antibiotic/Antimicrobial Resistance. http://www.cdc.gov/drugresistance/. Accessed on Februrary 25, 2016.

[3] Muto CA (2005). Why are antibiotic-resistant nosocomial infections spiraling out of control?. Infect Control Hosp Epidemiol.

[4] Samanipour A, Dashti-Khavidaki S, Abbasi M-R, Abdollahi A (2016). Antibiotic resistance patterns of microorganisms isolated from nephrology and kidney transplant wards of a referral academic hospital. J Res Pharm Pract.

[5] Sarnak MJ, Jaber BL (2000). Mortality caused by sepsis in patients with end-stage renal disease compared with the general population. Kidney Int.

[6] Krzowska-Firych J, Kozłowska A, Sukhadia T, Al-Mosawi LK. Hospital-acquired infections caused by antibiotic resistant bacteria. Postępy Nauk Medycznych. 2014.

[7] The state of the world’s antibiotic 2015. http://cddep.org/sites/default/files/swa_2015_final.pdf. Accessed on March 13, 2016.

[8] Performance Standards for Antimicrobial Susceptibility Testing; Twenty-Fourth Informational Supplement. http://ncipd.org/control/images/NCIPD_docs/CLSI_M100-S24.pdf. Accessed on January 10, 2016.

[9] Jones ME, Draghi DC, Thornsberry C, Karlowsky JA, Sahm DF, Wenzel RP (2004). Emerging resistance among bacterial pathogens in the intensive care unit–a European and North American Surveillance study (2000–2002). Ann Clin Microbiol Antimicrob.

[10] Anvarinejad M, Pouladfar G, Japoni A, Bolandparvaz S, Satiary Z, Abbasi P (2015). Isolation and antibiotic susceptibility of the microorganisms isolated from diabetic foot infections in Nemazee hospital, Southern Iran. J Pathog.

[11] Khalili H, Dashti-Khavidaki S, Shahidi MR, Abdollahi A, Jafari S, Jahangard-Rafsanjani Z (2012). Changes in gram negative microorganisms’ resistance pattern during 4 years period in a referral teaching hospital; a surveillance study. Daru.

[12] Moradi N, Javadpoor S, Vahdani M (2015). Prevalence and antibiogram pattern of gram negative bacteria isolated from blood cultures in shahid mohammadi hospital, Bandar Abbas. J Prev Med.

[13] Ishii Y, Alba J, Kimura S, Yamaguchi K (2006). Evaluation of antimicrobial activity of β-lactam antibiotics by Etest against clinical isolates from 100 medical centers in Japan (2004). Diagn Microbiol Infect Dis.

[14] Said KB, Al-Jarbou AN, Alrouji M, Al-harbi HO (2014). Surveillance of antimicrobial resistance among clinical isolates recovered from a tertiary care hospital in Al Qassim, Saudi Arabia. Int J Health Sci (Qassim).

[15] Hadadi A, Rasoulinejad M, Maleki Z, Yonesian M, Shirani A, Kourorian Z (2008). Antimicrobial resistance pattern of gram-negative bacilli of nosocomial origin at 2 university hospitals in Iran. Diagn Microbiol Infect Dis.

[16] Japoni A, Vazin A, Davarpanah MA, Ardakani MA, Alborzi A, Japoni S (2011). Ventilator-associated pneumonia in Iranian intensive care units. J Infect Dev Ctries.

[17] Hassanzadeh P, Motamedifar M, Hadi N (2009). Prevalent bacterial infections in intensive care units of Shiraz University of medical sciences teaching hospitals, Shiraz, Iran. Jpn J Infect Dis.

[18] National center of Rational Use Drug. http://fdo.behdasht.gov.ir/index.aspx?siteid=114&pageid=45999. Accessed on February 25, 2016.

[19] Khoshbakht R, Salimi A, Aski HS, Keshavarzi H (2012). Antibiotic susceptibility of bacterial strains isolated from urinary tract infections in Karaj, Iran. Jundishapur J Microbiol.

[20] Keen PL, Montforts MH. Antimicrobial resistance in the environment. John Wiley & Sons; 2011.

[21] Fish DN. Urinary tract infections. In: Alldredge BK, Corelli RL, Ernst ME, Guglielmo BJ Jr, Jacobson PA, Kradjan WA, et al (editors). Koda-Kimble and Young’s applied therapeutics: the clinical use of drugs, 10th edition. Philadelphia: PA: Lippincott Williams & Wilkins; 2012:1600.

[22] Mansour A, Manijeh M, Zohreh P (2009). Study of bacteria isolated from urinary tract infections and determination of their susceptibility to antibiotics. Jundishapur J Microbiol.

[23] Japoni A, Gudarzi M, Farshad S, Basiri E, Ziyaeyan M, Alborzi A (2008). Assay for integrons and pattern of antibiotic resistance in clinical Escherichia coli strains by PCR-RFLP in Southern Iran. Jpn J Infect Dis.

[24] Farshad S, Ranijbar R, Japoni A, Hosseini M, Anvarinejad M, Mohammadzadegan R (2012). Microbial susceptibility, virulence factors, and plasmid profiles of uropathogenic Escherichia coli strains isolated from children in Jahrom, Iran. Arch Iran Med.

[25] Oplinger M, Andrews CO (2013). Nitrofurantoin contraindication in patients with a creatinine clearance below 60 mL/min: looking for the evidence. Ann Pharmacother.

[26] Rahbar M, Gra Agaji R, Hashemi S (2005). Nosocomial blood stream infections in Imam Khomeini Hospital, Urmia, Islamic Republic of Iran, 1999-2001. East Mediterr Health J.

[27] Poorabbas B, Mardaneh J, Rezaei Z, Kalani M, Pouladfar G, Alami MH (2015). Nosocomial Infections: Multicenter surveillance of antimicrobial resistance profile of Staphylococcus aureus and Gram negative rods isolated from blood and other sterile body fluids in Iran. Iran J Microbiol.

[28] Bosso JA, Haines ML, Gomez J (2013). Stable Susceptibility to Aminoglycosides in an Age of Low Level, Institutional Use. Infect Dis Ther.

[29] Hsueh PR, Chen WH, Luh KT (2005). Relationships between antimicrobial use and antimicrobial resistance in Gram-negative bacteria causing nosocomial infections from 1991–2003 at a university hospital in Taiwan. International journal of antimicrobial agents.

[30] Glupczynski Y, Delmée M, Goossens H, Struelens M (2001). Distribution and prevalence of antimicrobial resistance among gram-negative isolates in intensive care units (ICU) in Belgian hospitals between 1996 and 1999. Acta Clinica Belgica.

[31] Patzer J, Dzierzanowska D, Turner P (2002). Susceptibility patterns of gram-negative bacteria from a Polish intensive care unit, 1997–2000. Int J Antimicrob Agents.

[32] Kucukates E (2005). Antimicrobial resistance among Gram-negative bacteria isolated from intensive care units in a Cardiology Institute in Istanbul, Turkey. Jpn J Infect Dis.

[33] Kooti S, Motamedifar M, Sarvari J (2015). Antibiotic resistance profile and distribution of oxacillinase genes among clinical isolates of Acinetobacter baumannii in Shiraz teaching hospitals, 2012-2013. Jundishapur J Microbiol.

[34] Shoja S, Moosavian M, Peymani A, Tabatabaiefar MA, Rostami S, Ebrahimi N (2013). Genotyping of carbapenem resistant Acinetobacter baumannii isolated from tracheal tube discharge of hospitalized patients in intensive care units, Ahvaz, Iran. Iran J Microbiol.

[35] Mohajeri P, Farahani A, Feizabadi MM, Ketabi H, Abiri R, Najafi F (2013). Antimicrobial susceptibility profiling and genomic diversity of Acinetobacter baumannii isolates: A study in western Iran. Iran J Microbiol.

[36] Karlowsky JA, Draghi DC, Jones ME, Thornsberry C, Friedland IR, Sahm DF (2003). Surveillance for antimicrobial susceptibility among clinical isolates of Pseudomonas aeruginosa and Acinetobacter baumannii from hospitalized patients in the United States, 1998 to 2001. Antimicrob Agents Chemother.

[37] Yaman A, Tasova Y, Kibar F, Inal AS, Saltoglu N, Buyukcelik O (2004). Investigation of the antibiotic susceptibility patterns of pathogens causing nosocomial infections. Saudi Med J.

[38] Vakili B, Fazeli H, Shoaei P, Yaran M, Ataei B, Khorvash F (2014). Detection of colistin sensitivity in clinical isolates of Acinetobacter baumannii in Iran. J Res Med Sci.

[39] Salehifar E, Shiva A, Moshayedi M, Kashi TS, Chabra A (2015). Drug use evaluation of Meropenem at a tertiary care university hospital: a report from Northern Iran. J Res Pharm Pract.

[40] Mahini S, Hayatshahi A, Torkamandi H, Gholami K, Javadi M (2015). Carbapenem utilization in critically Ill patients. J Pharm Care.

[41] Sistanizad M, Kouchek M, Miri M, Goharani R, Solooki M, Ayazkhoo L (2013). Carbapenem restriction and its effect on bacterial resistance in an intensive care unit of a teaching hospital. Iran J Pharm Res.

[42] Safari M, Saidijam M, Bahador A, Jafari R, Alikhani MY (2013). High prevalence of multidrug resistance and metallo-beta-lactamase (MβL) producing Acinetobacter baumannii isolated from patients in ICU wards, Hamadan, Iran. J Res Health Sci.

[43] Shahcheraghi F, Abbasalipour M, Feizabadi M (2011). Isolation and genetic characterization of metallo-β-lactamase and carbapenamase producing strains of Acinetobacter baumannii from patients at Tehran hospitals. Iran J Microbiol.

[44] Karmostaji A, Peerayeh SN, Salmanian AH (2013). Distribution of OXA-type class D β-lactamase genes among nosocomial multi drug resistant Acinetobacter baumannii isolated in Tehran hospitals. Jundishapur J Microbiol.

